# Identifying potential paraben transformation products and evaluating changes in toxicity as a result of transformation

**DOI:** 10.1002/wer.10705

**Published:** 2022-04-12

**Authors:** Michael T. Penrose, George P. Cobb

**Affiliations:** ^1^ Department of Environmental Science Baylor University Waco Texas USA

**Keywords:** disinfection byproducts, parabens, wastewater

## Abstract

Parabens are a class of compounds often used as preservatives in personal care products, pharmaceuticals, and food. They have received attention recently due to findings that demonstrate estrogenic impacts and other adverse effects of parabens. Release into wastewater effluent is considered a major contributor to the spread of parabens into surface water. Current regulations in areas such as Japan, Europe, and Southeast Asia limit the concentrations of parabens that can be used in formulations but do not address concentrations discharged into waterbodies. Recent studies suggest that parent parabens are effectively eliminated by transformation during the wastewater treatment processes. Common tertiary treatments include ultrafiltration, chlorination, UV disinfection and ozonation. Ultrafiltration is used to remove solids before a disinfection step. Of the disinfection steps, ozonation is often the most effective at removing parabens. Not much is known about the toxicities of paraben transformation products. Of the transformation products, chlorinated parabens and PHBA are the most studied. Previous studies have shown that chlorinated parabens have greatly reduced estrogen agonistic activity when compared with the activity of parents. However, more recent studies have found that halogenated parabens actually have estrogen antagonistic activity. Further research involving chlorinated parabens could include other toxic endpoints. No known studies have evaluated adverse effects of oxygenated parabens. Parabens can interact with chlorine residues in the environment and form chlorinated products, this will occur at a faster rate during chlorination. Ozonation will oxidize parabens and UV disinfection can both oxidize and halogenate parabens. All studies determining potential transformation products have been done in laboratory settings or specific conditions. Further research is needed to determine if these transformations occur in situ.

**Practitioner Points:**

- Common chemical processes utilized by wastewater treatment facilities are effective at transforming parabens.
- Paraben transformation products are released in greater concentration in effluent than parent paraben compounds.
- Halogenated transformation products have been identified as estrogen receptor antagonists.

## Graphical abstract

The image shows the movement of parabens through water treatment and into surface water. Primary treatments include clarifiers used for settling, secondary treatments include biological tanks, and tertiary treatments include both physical processes, such as ultrafiltration and UV disinfection and chemical processes, such as chlorination and ozonation. Parent paraben compounds will be transformed during biological and chemical treatments. Common transformation products include dichlorinated paraben species and PHBA.

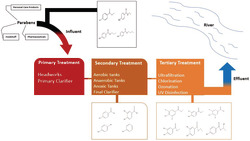

## INTRODUCTION

Personal care products (PCPs) are commonly used in products such as sunscreens, deodorants, shampoos, toothpaste and skin care products. Most personal care products are not regulated, and there are currently no limits for PCPs or pharmaceuticals in wastewater effluent (Yamamoto et al., 2011). Products used as coloring agents are currently the only products under the personal care product umbrella that are regulated by the FDA. Many of these PCPs go down our drains and into wastewater. Commonly used PCP ingredients such as preservatives, UV filters, coloring agents, and fragrances are organic and tend to be lipophilic. Many lipophilic organics have a greater ability to be absorbed via contact with skin, once adsorbed, a compound's properties will determine whether it is metabolized and excreted or if it bioaccumulates (Bledzka et al., 2014). UV filters and preservatives are both groups of stable compounds that can be used to increase product lifetime. Many organic preservatives include delocalized often aromatic structures that contribute to the compound's stability. Delocalized structures have charges distributed over a larger area rather than having one or a few atoms bearing the full weight of the charge. Compounds with localized charges are likely to have interactions at those charged sites. Stable compounds are more likely persist as they travel downstream and pass through primary and secondary wastewater treatment with little removal. Unstable products formed by degradation in the environment can form ROS species that would both facilitate the degradation of other ingredients used in the PCP as well as introduce harmful ecological impacts. Although preservatives are added to PCP formulations to prevent bacterial growth, parabens also improve the stability of the mixture (Nash & Tanner, 2014). This review intends to compare the effectiveness of different tertiary treatments on parabens, a class of antimicrobials that are often used mostly in personal care product formulations but can also be found in foods and pharmaceuticals. This paper will identify commonly used paraben compounds and give an overview of impacts of commonly used tertiary wastewater treatments on parabens, including possible disinfection products expected after treatment and toxicity of parabens and any evaluated transformation products. Removal rate is used as an umbrella term that combines changes in concentration from all routes into one percentage. This does not differentiate between removal by filtration, sorption, biodegradation, environmental degradation, or disinfection. In most cases, removal rate is determined for individual treatments and assumes that all observed changes in concentration result from a specific treatment. The term transformation products will be used to describe all products that are formed from of any type of reaction. In disinfection processes, most transformation products will be disinfection byproducts. Disinfection byproducts are any products that are formed during a disinfection process.

**FIGURE 1:**
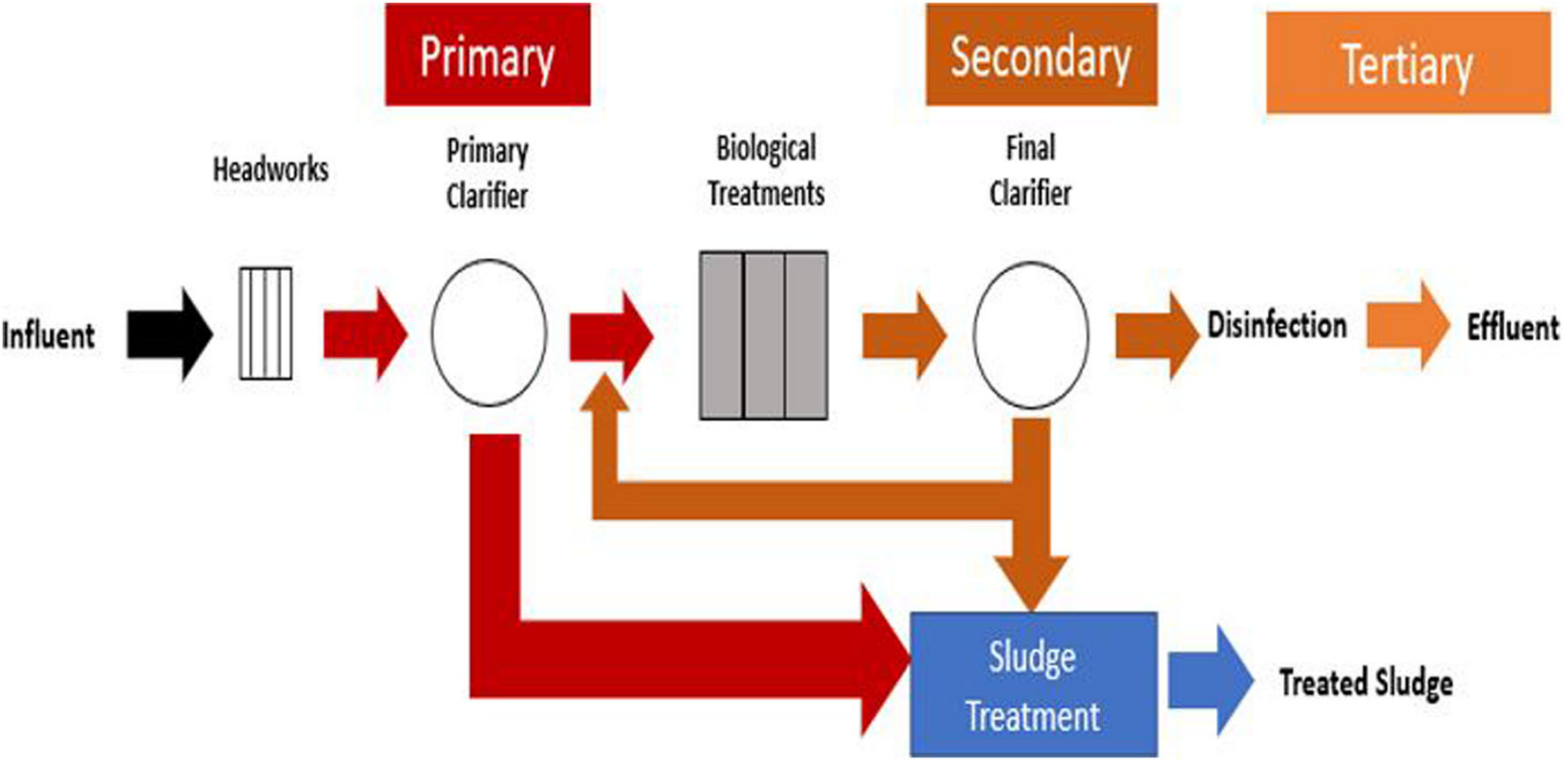
General water treatment process at water resource recovery facilities

Wastewater effluent is a major contributor to the spread of personal care products through surface waters (Brausch & Rand, 2011). A general setup includes primary, secondary and tertiary treatments (Figure 1). Primary treatment uses a settling chamber to reduce flow and allow for settling, secondary treatment uses microbiota to degrade material (Li et al., 2015b). Tertiary treatments could utilize both physical and chemical methods. The purpose of this manuscript is to cover previous research describing removal and toxicity and to determine potential transformation products while highlighting gaps in research in areas such as, transformation products in wastewater treatment, transformation products toxicity and transformation product persistence while comparing different commonly used tertiary treatments.

## PARABENS OVERVIEW

**FIGURE 2:**
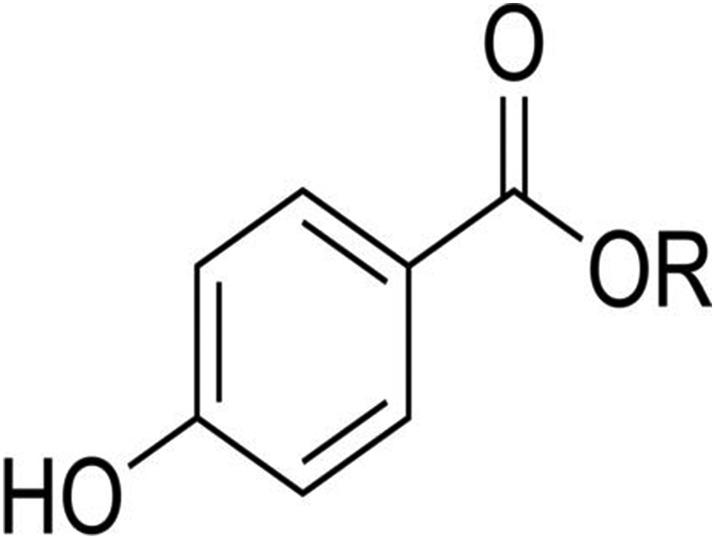
General paraben structure

Parabens are a class of compounds that all share para‐hydroxybenzoic acid (PHBA) as a structural component, often with an alkyl or benzyl group attached (Haman et al., 2015) (Figure 2). All parabens follow this trend with the main difference being in the alkyl chain attached to the carboxylic acid. For parent compounds, the chain only varies by number of carbons and hydrogens. Parent species is a term used to specify the original compound that has not yet been transformed or degraded. Not only are parabens effective preservatives, they also are chemically stable, cost effective and safe to use (Oishi, 2002). Methyl (MeP), ethyl (EtP), propyl (PrP), and butyl (BuP) parabens are more common parabens. Of the four, methyl and propyl paraben are used most often. Paraben regulations vary by agency. In Europe, the total concentration of all parabens in personal care products must not exceed 8 g of parabens per kg of product with the concentration of any individual parabens not to exceed 4 g of paraben per kg of product. In the United States, parabens have been designated as generally recognized as safe (GRAS) and can be used in products without approval by the FDA as long as no adverse effects have been seen when the product is used as intended. The Joint Expert Committee on Food Additives (JECFA) recommends limiting the maximum daily intake for parabens to 10 mg/kg of body weight. The JECFA also recommends that propyl paraben be excluded from use in foods (Sun et al., 2016). Parabens are ubiquitous, having been detected in most environments all around the globe. They can be released into the environment both directly and indirectly. Due to their use in PCPs, parabens can enter surface water directly by being desorbed/solubilized during recreational activities and can enter indirectly when present in wastewater effluent (Sharifan et al., 2016). Parabens have recently gained public attention due to findings that highlight different adverse effects ranging from oxidative damage to estrogenic impacts (Kasprzyk‐Hordern et al., 2008; Oishi, 2001). Paraben toxicity has been shown to increase as lipophilicity increases (Bledzka et al., 2014). Lipophilicity increases with increasing ester chain length, which also generally increases stability. Commonly used parabens include methyl, ethyl, propyl, butyl paraben, and parabens (Terasaki, Yasuda, & Shimoi, 2015). Benzyl paraben, isopropyl paraben, isobutyl paraben, and sec‐butyl paraben have been seen in smaller amounts; parabens with pentyl alkyl chains or longer are rarely used. In the environment, these compounds are likely to degrade or interact with environmentally available compounds.

## TRANSFORMATION OF PARABENS IN THE ENVIRONMENT AND EARLY STAGES OF WWTPS

**FIGURE 3:**
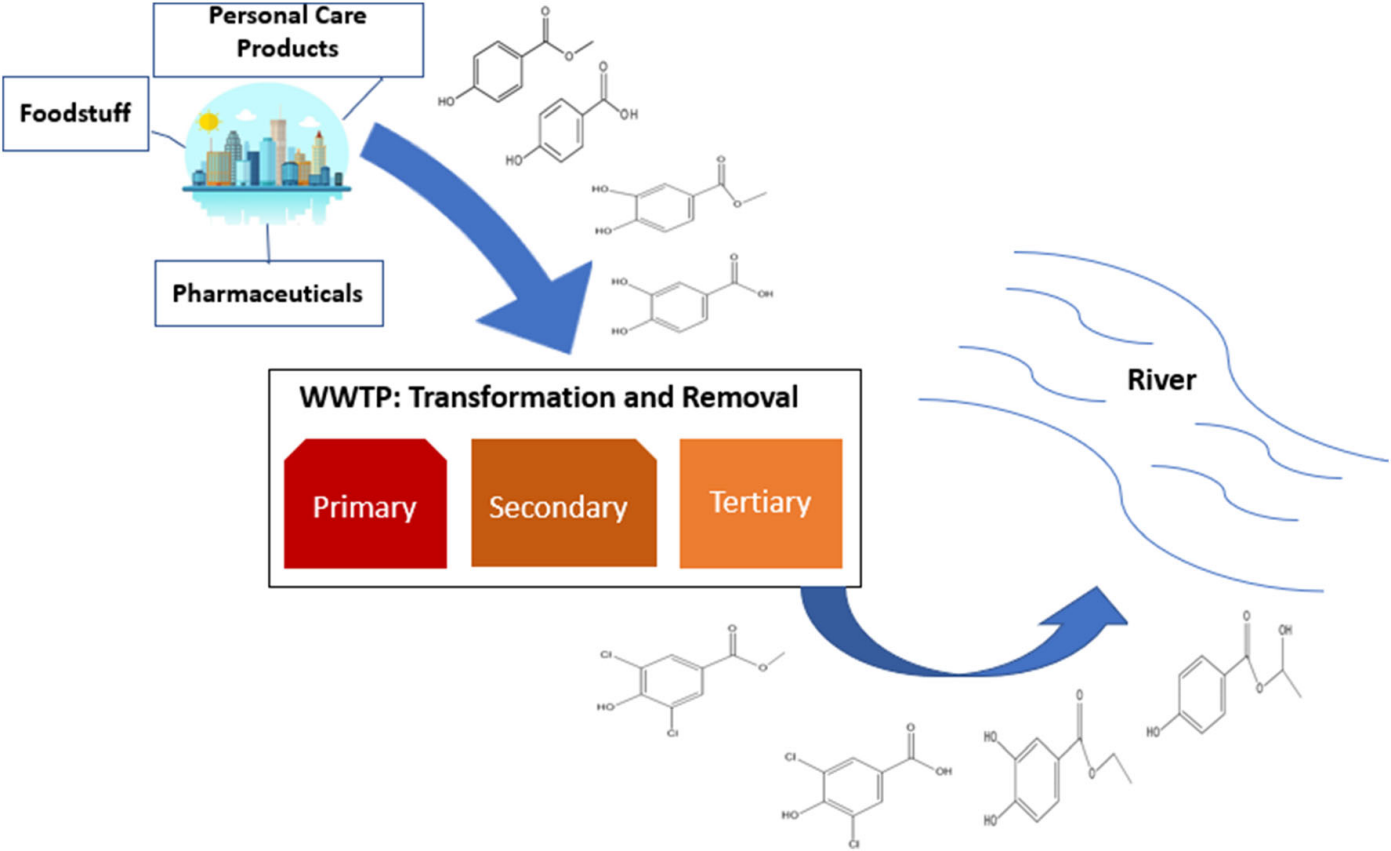
Movement of parabens from homes through paraben use in commercial products to the environment and from industry to the environment

The release of paraben transformation products in effluent would depend on both products entering the facility and the treatments used. Para‐hydroxybenzoic acid (PHBA) would be expected at intake, as the parent products are known to degrade in water. Transit times from point of use to the WWTP will allow degradation to proceed. Mono and di‐chlorinated products are also often seen in intake, as they are likely to form from free chlorine remaining after drinking water treatment. Brominated products may also be expected at intake. These are not as likely to form from transformation during treatment. However, they can form in the environment from bromine residues that are oxidized into HOBr, similarly to residual chlorine. Brominated products of parabens have been detected in river waters (Gouukon et al., 2020). Figure 3 shows the movement of parabens in personal care products from industry to the environment and from home use of commercial products.

Parabens are first introduced into the environment after being used in personal care product formulations, the products are applied and are eventually washed off the skin and down the drain in houses. Parabens then enter water systems and move towards treatment facilities. Before reaching wastewater treatment compounds have time to interact in the ambient environment, often being hydrolyzed into PHBA or interacting with free chlorine. Transformation products formed from these parent compounds during the wastewater process would depend on the treatments used. Usually, wastewater treatment includes primary treatment composed of clarifiers followed by a secondary treatment consisting of either anaerobic or anaerobic tanks degradation tanks. In primary treatments, there is change in paraben concentrations (Li et al., 2015b). Therefore, the only possible transformation products expected to form are the same as those seen formed in the environment by environmental degradation due to exposure to light or from hydrolysis in basic conditions. All parabens have a pK_a_ ranging from 8.2 to 8.6, and base hydrolysis will happen slowly even in more basic environments (Valkova et al., 2001). Paraben degradation in the environment occurs primarily by photodegradation with little occurring by hydrolysis, with studies finding that hydrolysis rates are too slow to be determined when working with environments below a pH of 10 (Xu et al., 2021). All the parent compounds share PHBA as their major degradation product, and there would be an expected increase in PHBA during these treatments. Degradation would most likely come from either interaction with microorganisms or through photodegradation. Mechanisms of photodegradation include the addition of hydroxyl radicals to the aromatic ring, and the abstraction of hydrogens (Gao et al., 2016). The addition of hydroxyls would form compounds such as hydroxyl monohydroxy‐methyl paraben. Paraben photolysis into PHBA and further degradation to hydroquinone and phenol can also occur (Gomes et al., 2017). In secondary treatments, activated sludge facilitate biotransformations, whereby esterases transform paraben into PHBA, and decarboxylase enzymes transform PHBA into phenol (Lu et al., 2018; Wu et al., 2016). Potential biodegradation efficiencies vary greatly by microorganism studied, compound, and conditions.

## REMOVAL OF PARABENS IN WASTEWATER TREATMENT

Most studies involving improving removal rates and determination of transformation products have been conducted in the laboratory and in specific environmental conditions. To our knowledge, published studies that evaluate removal rate focus on removal rate of parent parabens with a few quantifying removal of transformation products but do not study transformations that occur during treatment (Li et al., 2015a; Ma et al., 2018; Song et al., 2017; Wang & Kannan, 2016). Laboratory conditions all involved controlled temperatures, pH, and concentrations (Dhaka et al., 2017; Gomes et al., 2017; Mao et al., 2016; Tay et al., 2010a). The specific environmental conditions include a study over volcanic rocks and a study involving evaluating transformation in swimming pools (Gomes et al., 2017; Li et al., 2015a). The study involving volcanic rocks is a laboratory study setting up a volcanic environment and adding ozone to determine if volcanic rocks impact ozonation of parabens, meaning that the study included both environmental conditions as well as a controlled laboratory environment. The study by Gomes et al. (2017) evaluated ozonation over volcanic rocks by using rocks collected from São Miguel and evaluated paraben ozonation with a photoreactor and ozone. The study also included controlled temperatures and ozone concentrations. The study by Li et al. (2015b) evaluated parabens and chlorinated byproducts in swimming pool water. However, this study did not directly evaluate transformation of parabens into chlorinated transformation products, but rather evaluated the presence of chlorinated products that are produced from parabens in the chlorinated environment. Removal of organics will vary based on the size of the chemical and its physiochemical properties. Longer chained organics are often more slowly transformed, often due to increasing chemical stability (Haman et al., 2015). Organics are mostly removed by tertiary treatments. Removal methods can be broken up into two types, physical methods, and chemical methods. Ultrafiltration is a physical removal method used in tertiary treatment. Unlike the other methods that will be covered, ultrafiltration is exclusively a separation method and not a disinfection process. UV disinfection is a physical disinfection method, whereas chlorination and ozonation are chemical disinfection methods. UV disinfection does not add chemical reagents to the water, instead transformation will occur due to residues and compounds already present in water flowing through the treatment facility (Li et al., 2015b). Dichlorinated paraben species can be found in intake, likely due to residual chlorine and high persistence once formed. Monochlorinated species, PHBA and hydroxylated species have also been found at intake. Of the transformation products, only chlorinated and hydroxylated forms of methyl and ethyl parabens have been quantified, no longer chained species have been evaluated in wastewater. Studies that emphasize the treatment of parabens species in wastewater tend to focus on the overall effectiveness of the treatment, which includes removal of the paraben through sorption, filtration, and transformation in order to determine. In this type of assessment, the fraction of the parent compound remaining after treatment is determined (Li et al., 2015b). However, no research has been done to quantify changes in transformation product concentrations during tertiary treatments. Transformation products have been evaluated in laboratory settings under specific conditions. Three studies have evaluated the removal of both parabens and select transformation products; these studies evaluated wastewater facilities located in the United States (Wang & Kannan, 2016), China (Ma et al., 2018), Spain (Albero et al., 2012), and India (Karthikraj et al., 2017). These articles mainly evaluated the removal of parabens and metabolites found in intake and not on products formed during treatment. These studies have shown that parent parabens are well removed in wastewater treatment. Wang and Kannan (2016) evaluated removal of six parent parabens (methyl paraben, ethyl paraben, propyl paraben, butyl paraben, heptyl paraben, and benzyl paraben) and five metabolites (benzoic acid, PHBA, DHBA, hydroxymethyl paraben, and hydroxyethyl paraben) in two wastewater treatment plants utilizing biological treatments. Results of the study indicated that parent compounds are removed more effectively than metabolites with average removal efficiencies of parent compounds ranging from 70.8% to 96.6% and metabolite removal efficiencies ranging from 61.4% to 90.6%. Ma et al. (2018) evaluated the same paraben and metabolites as did Wang and Kannan (2016) in two treatment facilities that utilized A/O (anaerobic‐oxic) and CAST (cyclic activated sludge technology) treatments and found that metabolite removal efficiencies were higher, with an overall metabolite removal of 98.4% as compared with 81.6% for parent parabens. Albero et al. (2012) determined removal efficiencies for five parent parabens (methyl, ethyl, propyl, butyl, and benzyl paraben) by quantifying the parent compounds in influents and effluents of 19 different water resource recovery facilities. Paraben compounds had removal efficiencies at greater than 90% in chlorination, biological treatments, and sludge treatments. Karthikraj et al. (2017) found that removal efficiencies for parent compounds from five different treatment facilities ranged from 90% to 100% whereas metabolites ranged from 28% to 76%. The combined paraben and metabolite concentrations ranged from 2610 to 3820 ng/L with metabolites comprising 93%–99.6% of the total concentration (Karthikraj et al., 2017). Li et al. (2015b) evaluated removal efficiencies of eight parent parabens (methyl paraben, ethyl paraben, propyl paraben, butyl paraben, pentyl paraben, heptyl paraben, benzyl paraben, and octyl paraben), PHBA and four chlorinated transformation products (methyl 3‐chloro‐4‐hydroxybenoate, ethyl 3‐choloro‐4‐hydroxybenzoate, methyl 3,5‐dichloro‐4‐hydroxybenzoate, and ethyl 3,5‐dichloro‐4‐hydroxybenzoate) at a water resource recovery facility in China that utilized biological tanks, ultrafiltration and ozonation. Parent compounds were well removed at 97.6% to 99.8% removal efficiencies, PHBA had a decent removal efficiency at 78.6% whereas the two dichlorinated transformation products had low removal efficiencies with 40.7% removal for dichlorinated methyl paraben and 33.9% for dichlorinated ethyl paraben. When comparing the different studies, parent compounds generally had higher removal rates than that of the metabolites/transformation products. However, there could be some complication due to the potential formation of transformation products throughout treatment that were not accounted for in these studies. Although laboratory studies have evaluated formation of longer chained hydroxylated and chlorinated parabens, no studies have quantified them in wastewater treatment or in the environment possibly due to lack of standards for these compounds (Albero et al., 2012). Overall biological treatments and tertiary chemical treatments had the greatest impact on paraben removal. However, not a large amount of the removal is expected to occur by settling or sludge treatment during primary treatment suggesting that much of the removal occurs due to transformation of the parent compounds either through biodegradation in biological tanks or through chemical treatments (Song et al., 2017). Parent parabens have been detected at low concentrations in sludge. In a study by Karthikraj et al. (2017), of the parent paraben compounds, methyl paraben had been quantified in highest concentrations at 176.2 to 339 ng/L which made up 59%–79% of total paraben concentration. All other parent compounds were detected at much lower concentrations. A study by Li et al. (2015b) determined that sorption to sludge accounted for less than 7.5% removal of total parabens. There have been no known studies that address transformations that occur at different treatment steps. This demonstrates a need to evaluate transformation products formed during the wastewater treatment process.

### Removal by chlorination

Chlorination is the older disinfection method and is more often used for drinking water treatment rather than for wastewater treatment. Chlorination usually uses involves the use of chloramine, which is an amine with a chlorine attached. However, chlorine gas can be used as well. Both will react with water to form hypochlorous acid (HOCl) and hydrochloric acid (HCl) both of which have the ability to chlorinate compounds. HOCl can have an oxidative reaction as well. Due to the use of chlorination in drinking water treatment, chlorine containing products are often found in wastewater influent. PHBA and other formed products generally have lower removal rates than that of parent species. Chlorinated species are generally more stable than their parent compound leading to greater persistence in the environment. If previously transformed products are not included, chlorination has a 70% removal rate of paraben species in wastewater (Mao et al., 2016). In many water systems, especially in more chlorinated systems, halogenated species are present in a greater amount than that of the parent compound or PHBA (Li et al., 2015b).

#### Disinfection byproducts expected from chlorination

Chloramine addition to water increases concentration of hypochlorous acid and hydrochloric acid. Hypochlorous acid dissociates into hypochlorite ions^−^, and hydrochloric acid disassociates resulting in a chloride ion. The most common chlorination mechanism by both formed chemicals involves the addition of a chlorine on the aromatic ring, potentially followed by a second addition (Yoom et al., 2018). The addition of chlorine can be followed by the addition of a bromine or vice versa, due to the hypobromous acid (HOBr) which may form from free bromine.

If the compound is not already chlorinated or brominated before chlorination, a chlorine will be added on the aromatic ring, followed quickly by a second addition (Table 1). Additional chlorines can be added in cases where paraben compounds are reacted with high concentrations of chlorine. However, products with additional chlorine will not form in wastewater treatment or from interaction with chlorine residues in normal environment situations. Wastewater treatment typically aims for a concentration of 1 to 15 mg of chlorine per liter of water during chlorination with an average contact time of 30 min. At 10 mg of chlorine per liter of water, further chlorination would take days (Yoom et al., 2018). A previously brominated paraben will act similarly to a previously chlorinated one, a monobrominated paraben will quickly react and form a compound with one chlorine and one bromine. A dibrominated paraben will most likely not react.

**TABLE 1:** Paraben reaction rates with chlorine (rates with 40 μM chlorine concentration determined by Mao et al., 2016, with 113 μM by Yoom et al., 2018)

Chemical	Number of chlorinations	Final chlorine concentration in solution	Concentration of paraben in solution	Rate
Methyl paraben	One	40 μM	2.6 μM	9.65 × 10^−3^ M^−0.614^ s^−1^
	One	113 μM	1 μM	64 M^−1^ s^−1^
	Two	113 μM	1 μM	243 M^−1^ s^−1^
	Three	113 μM	1 μM	1.3 M^−1^ s^−1^
Ethyl paraben	One	40 μM	2.4 μM	1.77 × 10^−2^ M^−1.019^ s^−1^
Propyl paraben	One	40 μM	2.2 μM	2.98 × 10^−2^ M^−0.851^ s^−1^
Butyl paraben	One	40 μM	2.1 μM	1.76 × 10^−2^ M^−0.860^ s^−1^

Hypochlorous acid formed during tertiary treatment may allow the hydroxylation of the ring, though increases in dihydroxy paraben concentrations are expected to be low compared with the increases expected in UV disinfection and ozonation. Degradation products, such as PHBA, and dichlorinated parabens are not likely to degrade further into other compounds in the absence of biological activity. Other transformation products including dichlorinated paraben species have lower removal rates than that of the parent species in ozonation and UV disinfection (Li et al., 2015b). However, past studies have evaluated dichlorinated paraben removal rather than monochlorinated. Monochlorinated parabens will be more likely to transform in wastewater treatment than the dichlorinated products. More research is needed to quantify the change in chlorinated paraben species after chlorination. Halogenation of paraben species would deactivate the aromatic ring resulting in decreased reactivity. Aromatic ring deactivation decreases further transformation of the dihalogenated species. Dichlorinated parabens degrade very slowly, and likely would not see significant changes due to natural degradation during the process. Therefore, it is necessary to determine if chlorinated parabens are released in greater concentrations after chlorination when compared with the concentrations that come in as influent. Dichlorinated methyl and ethyl paraben removal rates have been determined from ultrafiltration and ozonation. Currently, no known studies have quantified chlorinated parabens concentrations changes from influent water to concentrations in effluent after chlorination. There is likely to be an increase in chlorinated disinfection product concentration following a chlorination step. Products that can be expected from chlorination include mono and dichlorinated forms of all used parabens, mono and dichlorinated forms of PHBA (Table 2). In environments where higher concentrations of residual bromine, mixtures of chlorinated and brominated parabens could also form.

**TABLE 2:** Major disinfection byproducts expected after chlorination

Transformation product	Chemical formula	Structure	Molecular weight (g/Mol)
3‐Chloro‐methyl paraben	C_8_H_7_O_3_Cl	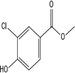	186.59
3‐Chloro‐ethyl paraben	C_9_H_9_O_3_Cl	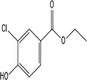	200.62
3‐Chloro‐propyl paraben	C_10_H_11_O_3_Cl	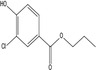	214.65
3‐Chloro‐butyl paraben	C_11_H_13_O_3_Cl	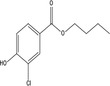	228.67
3‐Chloro‐PHBA	C_7_H_5_O_3_Cl	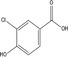	172.57
3,5‐Dichloro‐methyl paraben	C_8_H_6_O_3_Cl_2_	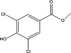	221.04
3,5‐Dichloro‐ethyl paraben	C_9_H_8_O_3_Cl_2_	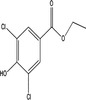	235.06
3,5‐Dichloro‐propyl paraben	C_10_H_10_O_3_Cl_2_	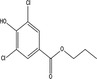	249.09
3,5‐Dichloro‐butyl paraben	C_11_H_12_O_3_Cl_2_	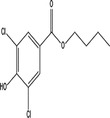	263.12
3,5‐Dichloro‐PHBA	C_7_H_4_O_3_Cl_2_	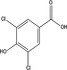	207.01
5‐Chloro‐DHBA	C_7_H_4_O_4_Cl	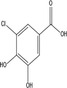	187.56

### Removal by ultrafiltration

The purpose of ultrafiltration is to remove larger solids from water for cleaner effluent. Large particles could otherwise obstruct the disinfection step that follows. Ultrafiltration works by separating biota, and solids from water based on size. Pressure is often applied to push water through along with small molecules. However, the pore size used in ultrafiltration is usually from 2 to 100 nm, often too large to exclude small compounds on its own (Li et al., 2015b). If parabens aggregate on a sediment particle, the paraben‐particle assemblage may be removed by the filter.

How well a chemical is filtered depends on how strongly the chemical would sorb to suspended solids or size of the compound, if the analytes of interest are large and the filter pore size is very small, then size could play a role in removal. If a compound is likely to desorb then a large percentage will be soluble in the water and will not be removed as water pushed through the filter. Larger chlorinated transformation products are generally more likely to sorb to sediments than are their untransformed parent compounds. Thus, chlorinated parabens are expected to have higher removal rates using ultrafiltration The difficulty in this method lies in the large pore size, with ultrafilters used in wastewater treatment intended to remove larger material that is greater than 5 kDa in weight. However, smaller pores would be harder to get and more expensive. Because water needs to get through, there is a lower limit of pore size. A water molecule is about 0.275 nm.

Table 3 demonstrates the removal of parent parabens and chlorinated transformation products in a general wastewater treatment setup that include ultrafiltration and ozonation. Ultrafiltration had a very low removal rate for parent compounds, and though the rate was higher for chlorinated products, the removal rates are still very low compared with other treatments (Li et al., 2015b). However, it is important to note that ultrafiltration removes larger solids from wastewater. Solids would reduce the effectiveness of ozonation and UV disinfection, both of which need more access to the chemical.

**TABLE 3:** Removal efficiency of specific paraben species by ultrafiltration and ozonation (Li et al., 2015b)

Compound	Chemical formula	Molecular weight (g/Mol)	Removal efficiency (%)	
			Ultrafiltration	Ozonation
Methyl paraben	C_8_H_8_O_3_	152.15	<1	98.8
Ethyl paraben	C_9_H_10_O_3_	166.17	<1	99.8
Propyl paraben	C_10_H_12_O_3_	180.2	<1	99.9
Butyl paraben	C_11_H_14_O_3_	194.23	<1	99.7
3,5‐Dichloro‐methyl paraben	C_8_H_6_O_3_Cl_2_	221.04	9.9	82.8
3,5‐Dichloro‐ethyl paraben	C_9_H_8_O_3_Cl_2_	235.06	3.1	59.2

The chlorinated parabens were removed by ultrafiltration at higher rates than any of the parent parabens, likely due to sorption to suspended solids (Li et al., 2015b). The chlorinated species would be more likely to sorb to suspended sediments, which would allow removal if the compounds do not desorb by water flowing through. Of the chlorinated species, the dichlorinated methyl paraben was removed at a greater efficiency than ethyl paraben. The larger sizes of the chlorinated species may also play a minor role in removal if including the largest parabens such as chlorinated benzyl parabens. In this case with the methyl and ethyl compound, the halogenated species are still too small to be filtered, especially considering the larger compound was the one with the lower removal efficiency. The ethyl compound would have a greater affinity to suspended solids. Therefore, the results showing that the methyl compound had a greater removal rate was unexpected. However, the ethyl species is more stable. Perhaps the differences in efficiency could be related to factors other than ultrafiltration, such as degradation in the water or when sorbed to suspended sludge. Because ultrafiltration does not directly introduce chemicals or facilitate radical formation for reactions, any increases in transformation product concentrations would not be directly related to the treatment.

### Removal by ozonation

Ozonation is a newer disinfection method that is becoming the more popular method. Ozonation works by pumping ozone (O_3_) into wastewater, which can result in an addition of a hydroxyl group to the aromatic ring or can be added to the chain on compounds with longer chains among other reactions (Xiao et al., 2015). Removal efficiencies vary by chain length (Table 3).

Ozone reactions will vary based on environmental factors such as, the amount of light and presence of solids. In the presence of light ozone can form hydroxyl radicals that will interact with organic compounds to form an oxidized transformation product. Ozone could also react with water and form hydrogen peroxide (H_2_O_2_) which can breakdown into hydroxyl radicals to interact with organic compounds (Xiao et al., 2015).

Ozonation can cause transformation through three mechanisms. The first mechanism involves the formation of hydroxyl and superoxide radicals that then interact with parabens (Tay et al., 2010b). Another mechanism involves the direct oxidation of compound by ozone (Xiao et al., 2015). Although the oxidation by hydroxyl radicals is nonselective direct oxidation by ozone is selective and requires double bonds. Direct oxidation by ozone begins with the interaction between ozone and a carbon on the aromatic ring of a paraben. The double bond is broken, forming an unstable cation. Instead of completing lysis, the double bond reforms (Tekle‐Rottering et al., 2016). The final mechanism is ozonolysis, this mechanism is not expected to occur with paraben compounds as this could only occur at one of the double bonds on the aromatic ring, bond breakage on a stable delocalized structure, such as aromatic rings would be unfavorable. Ozonation can cause an addition of a hydroxyl group to the aromatic ring. This method often yields high rates of organic transformations, generally removing anywhere from 85% to 99% of depending on the compound (Li et al., 2015b). The parent paraben species had high removal rates with methyl parabens having the lowest removal rate. Methyl paraben would be expected to have the highest removal rate due to having the shortest chain. However, longer chained species could degrade to the shorter chain species. This has been seen to occur from paraben degradation in oxidative environments (Argenta et al., 2020). Transformation from a longer chained paraben to methyl paraben would cause difficulties in determining transformation rates. Methyl paraben will have a lower transformation and removal rates because there is no easy way to distinguish between methyl paraben concentration increases from longer chained degradation and untransformed methyl paraben. This should be considered in other treatments as well, larger chained parabens could potentially undergo natural degradation to methyl paraben in other treatments that could skew data to imply lower removal for rate for methyl parabens, especially in facilities with an open system. Chlorinated parabens species have lower removal rates than that of parent paraben species. This is something to consider for all wastewater treatments. The dichlorinated methyl and ethyl parabens had an ozonation removal efficiency of 82.8% and 59.2% respectively. These followed the expected trend, with the longer chained compound having a lower removal rate. No known studies have evaluated removal of longer chained chlorinated paraben species. Parent products and expected chlorinated products are included in Table 2. Oxidized disinfection products will be more resistant to further oxidation (Tay et al., 2010a).

Direct oxidation with ozone is impacted by the pH of the surrounding environment. Reaction rates for ozone interaction with methyl ethyl and butyl parabens at a pH of 6 were 1000 times higher than the reaction rates at a pH of 2 and rates at a pH of 12 was 10^7^ times higher (Tay et al., 2010b). Reaction rates for the oxidation of parabens by hydroxyl radicals are higher than reaction rates for oxidation by ozone with reaction rates between 10^9^ for methyl paraben and 10^10^ M^−1^ s^−1^ for butyl paraben when in an aqueous solution at a pH of 9.9, and rates for oxidation by ozone at a pH of 12 range from 1.02 × 10^9^ for methyl paraben to 1.38 × 10^9^ M^−1^s for butyl paraben^−1^ (Tay et al., 2010b). The most common transformation products expected from ozonation are dihydroxylated parabens, as well as PHBA and hydroquinone. However, hydroquinones are likely to form from many other products that may be found wastewater and is used in as well. Therefore, it would be difficult to determine if increases in hydroquinone concentration are specifically a result of paraben transformation during treatment. The hydroxyl radical can also facilitate hydrolysis of paraben into PHBA. Common mechanisms for the interaction of parabens with hydroxyl radicals include the addition of one hydroxyl radical and the abduction of a hydrogen. These mechanisms are discussed in greater detail in Gao et al., 2014 and Gao et al., 2016. Transformation products from ozonation will include dihydroxylated species, formed by direct oxidation by ozone and by hydroxyl radical formation. Further transformations by hydroxylation are less likely to occur. Degradation by hydroxyl radicals will form PHBA. PHBA in influent will be oxidized to 3,4‐dihydroxybenzoic acid (DHBA). The toxicity of oxidized parabens has not been studied. Research conducted on ozonation in laboratory settings has detected dihydroxylated and trihydroxylated parabens, as well as hydroquinones, phenols and benzoic acid (Dhaka et al., 2017; Gomes et al., 2017; Tay et al., 2010b). Direct oxidation by ozone can only add hydroxyl groups on the aromatic ring as direct oxidation by ozone requires double bonds. Benzyl paraben has a second aromatic ring as a side chain. Therefore, direct oxidation by ozone could occur on the side chain for benzyl paraben. Oxidation by hydroxyl radicals, on the other hand, can add groups to the side chains of longer chained parabens, addition on the alkyl chain has been seen with parabens of varying chain length but occurs more frequently on longer chained parabens (Tay et al., 2010b) (Table 4).

**TABLE 4:** Major paraben transformation products detected in laboratory studies from interaction with ozone and hydroxyl radicals

Transformation product	Chemical formula	Structure	Molecular wt. (g/Mol)	Experimental conditions
Phenol^b^	C_6_H_6_O	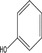	94.11	Temperature: 25°C pH: 6.5 UV lamps (254 nm): Radical production Paraben concentration: 19.5–65.7 μM Persulfate concentration: 0.5–4 mM
Hydroquinone^c^	C_6_H_6_O_2_	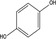	110.11	Temperature: 25°C UV lamps (256 nm) Paraben concentrations: 10 mg/L Catalyst concentration: 70 ng/L Catalysts (Pt‐TiO_2_, Pd‐TiO_2_, ag‐TiO_2_) Ozone concentration: Up to 120 mg/L
PHBA^a^	C_7_H_6_O_3_	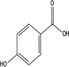	127.12	Temperature: 25°C pH: 2, 6, 12 Paraben concentration: 100–1000 μM Ozone dose: 0.43 to 0.86 g/h
DHBA^b^	C_7_H_6_O_4_	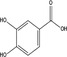	154.12	Temperature: 25°C UV lamps (256 nm) Paraben concentrations: 10 mg/L Catalyst concentration: 70 ng/L Catalysts (Pt‐TiO_2_, Pd‐TiO_2_, ag‐TiO_2_) Ozone concentration: Up to 120 mg/L
1‐Hydroxy‐methylparaben^a^	C_8_H_8_O_4_	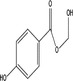	168.15	Temperature: 25°C pH: 2, 6, 12 Paraben concentration: 100–1000 μM Ozone dose: 0.43 to 0.86 g/h
Monohydroxy‐methyl paraben^a^	C_8_H_8_O_4_	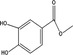	168.15	Temperature: 25°C pH: 2, 6, 12 Paraben concentration: 100–1000 μM Ozone dose: 0.43 to 0.86 g/h
Dihydroxy‐methyl paraben^a^	C_8_H_8_O_5_	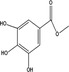	184.15	Temperature: 25°C pH: 2, 6, 12 Paraben concentration: 100–1000 μM Ozone dose: 0.43 to 0.86 g/h
Trihydroxy‐methyl paraben^a^	C_8_H_8_O_6_	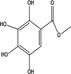	200.15	Temperature: 25°C pH: 2, 6, 12 Paraben concentration: 100–1000 μM Ozone dose: 0.43 to 0.86 g/h
Monohydroxy‐ethyl paraben^a^	C_9_H_10_O_4_	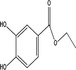	182.17	Temperature: 25°C pH: 2, 6, 12 Paraben concentration: 100–1000 μM Ozone dose: 0.43 to 0.86 g/h
1‐Hydroxy‐ethylparaben^a^	C_9_H_10_O_4_	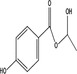	182.17	Temperature: 25°C pH: 2, 6, 12 Paraben concentration: 100–1000 μM Ozone dose: 0.43 to 0.86 g/h
1,2‐dihydroxy‐ethylparaben^a^	C_9_H_10_O_5_	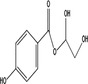	198.17	Temperature: 25°C pH: 2, 6, 12 Paraben concentration: 100–1000 μM Ozone dose: 0.43 to 0.86 g/h
Dihydroxy‐ethyl paraben^a^	C_9_H_10_O_5_	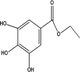	198.17	Temperature: 25°C pH: 2, 6, 12 Paraben concentration: 100–1000 μM Ozone dose: 0.43 to 0.86 g/h
Monohydroxy‐propyl paraben^a^	C_10_H_12_O_4_	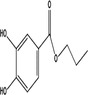	196.2	Temperature: 25°C pH: 2, 6, 12 Paraben concentration: 100–1000 μM Ozone dose: 0.43 to 0.86 g/h
Dihydroxy‐propyl paraben^a^	C_10_H_12_O_5_	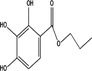	212.2	Temperature: 25°C pH: 2, 6, 12 Paraben concentration: 100–1000 μM Ozone dose: 0.43 to 0.86 g/h
Trihydroxy‐propyl paraben^a^	C_10_H_12_O_6_	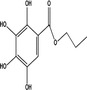	228.2	Temperature: 25°C pH: 2, 6, 12 Paraben concentration: 100–1000 μM Ozone dose: 0.43 to 0.86 g/h
1‐Hydroxy‐butylparaben^a^	C_11_H_14_O_4_	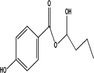	210.23	Temperature: 25°C pH: 2, 6, 12 Paraben concentration: 100–1000 μM Ozone dose: 0.43 to 0.86 g/h
4‐(hydroxybenzoyl oxy) butanoic acid^a^	C_11_H_12_O_5_	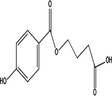	224.21	Temperature: 25°C pH: 2, 6, 12 Paraben concentration: 100–1000 μM Ozone dose: 0.43 to 0.86 g/h
1‐Hydroxy‐2‐oxobutyl 4‐hydroxybenzoate^a^	C_11_H_12_O_5_	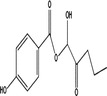	224.21	Temperature: 25°C pH: 2, 6, 12 Paraben concentration: 100–1000 μM Ozone dose: 0.43 to 0.86 g/h
Dihydroxy‐butyl paraben^a^	C_11_H_14_O_5_	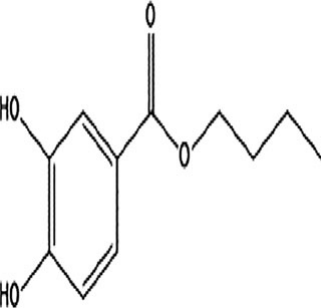	226.23	Temperature: 25°C pH: 2, 6, 12 Paraben concentration: 100–1000 μM Ozone dose: 0.43 to 0.86 g/h
Trihydroxy‐butyl paraben^a^	C_11_H_14_O_6_	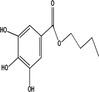	242.22	Temperature: 25°C pH: 2, 6, 12 Paraben concentration: 100–1000 μM Ozone dose: 0.43 to 0.86 g/h

^a^ Product determined by Tay et al. (2010b).

^b^ Product determined by Dhaka et al. (2017).

^c^ Product determined by Gomes et al. (2017).

### Removal by UV disinfection

**FIGURE 4:**
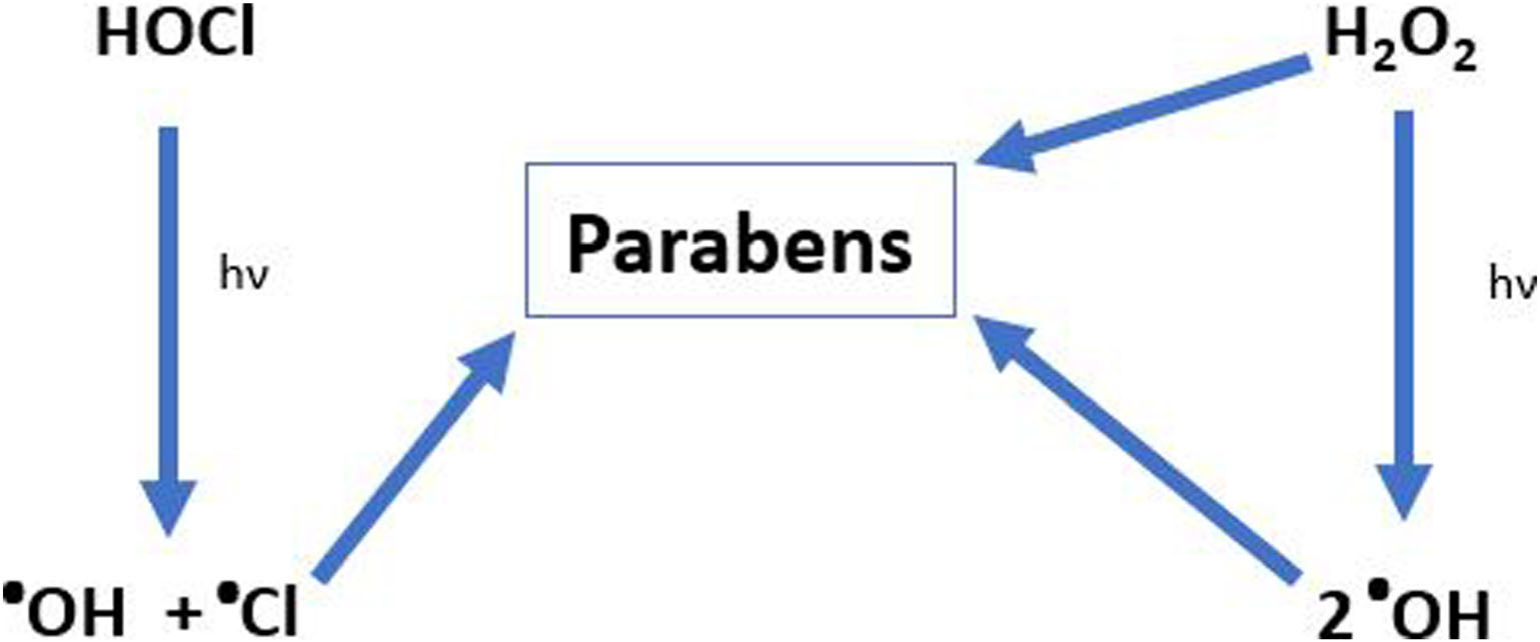
Formation of hydroxyl radicals during UV disinfection

UV irradiation is a commonly used disinfection method, due to efficient bacterial incapacitation and lack of residue formation. UV disinfection uses molecules already present in the ambient environment to transform compounds. This is usually done through the formation of radicals which can either oxidize the compound often resulting in an addition of a hydroxyl or hydrolysis to PHBA (Tay et al., 2010a; Zúñiga‐Benítez et al., 2018). UV light facilitates bond lysis, producing smaller molecules. Photolysis of paraben species will occur in the presence of light, with the primary product being PHBA (Gomes et al., 2018). Methyl paraben has maximum absorption at 254 nm. UV disinfection uses UVC lights which have wavelengths ranging from 200 to 280 nm. Therefore, direct excitation of paraben compounds is possible. Radicals will form from UVC light interacting with other compounds present in the environment. The excitation of parabens will also facilitate the interaction between paraben species and these radicals. Whether the reaction is more reliant on the excitation of parabens, or the formation of radicals is undetermined. If chloramine is present, UV light can cause the formation of chloride and hydroxyl radicals which will interact with compounds (Figure 4). Currently, many facilities utilize direct radiation using UV lamps. Studies have shown that catalyzed photolysis is more efficient at transforming compounds than direct photolysis (Gmurek et al., 2017).

Because UV degradation requires low amounts of suspended solids, ultrafiltration is often included just before treatment. UV disinfection can use titanium dioxide or iron catalysts to aid in hydroxyl radical formation. Titanium dioxide catalysts prove to aid in radical formation (Gomes et al., 2017). If an iron catalyst is used, hydrogen peroxide is commonly used to facilitate paraben‐radical interactions. A study by Frontistis et al. (2015) found that the combination of an iron catalyst and hydrogen peroxide was move effective at inducing photolytic degradation than when used alone and that UVC radiation is much more effective at inducing photodegradation when compared with solar light. This means that wastewater treatment facilities could improve their UV disinfection processes by adding a hydrogen peroxide along with a catalyst. Studies have found that methyl paraben is more resistant to degradation by UV light than other longer chained parent parabens (Foszpanczyk et al., 2018). This is similar to what was seen with ozonation, larger chain degradation into methyl paraben could also be a factor in the lower transformation rate.

#### Transformation products from UV disinfection

Most studies that focus on the impacts of UV irradiation on parabens, evaluate interaction under catalyzed conditions. In these cases, many different reactive species are formed such as hydroxyl radicals, superoxides and hydroperoxyl radicals (Van‐Huy Nguyen et al., 2021). Titanium dioxide (TiO_2_) is one commonly used catalyst to enhance paraben oxidation by UV radiation (Salimi et al., 2017). A study by Zúñiga‐Benítez et al. (2018) demonstrated the effects of a catalyst on the removal rates of parabens and other organics used in PCPs, use of a catalyst significantly increased removal rate. UV disinfection can produce many different products. By forming chorine and hydroxyl radicals, UV disinfection can generate both chlorinated and hydroxylated products. Concentrations of chlorinated transformation products fare expected to be less than concentrations quantified in chlorination, and the concentrations of oxidized paraben products will be less than concentrations expected from ozonation. Previous studies involving catalyzed UV degradation of parabens have identified different transformation products that arise from reaction with hydroxyl radical which will be able to add hydroxyl groups to both the side chain and the aromatic ring. Similarly, to ozonation major byproducts will include dehydroxylated paraben species and may include trihydroxylated species. Chlorinated products will be less prevalent than the hydroxylated products. Many wastewater treatments that use UV disinfection only use direct photolysis using UV lamps without catalysts. Although there have been studies involving recovery rates using direct photolysis, all known studies that identify transformation products resulting from UV degradation include catalysts. This is likely due to earlier results that indicate improved removal rates in the presence of catalysts improving radical formation. Therefore, research focusing on transformation product formation at wastewater treatment sites would be beneficial in identifying which of the products are actually being formed.

## PARABEN TOXICITY AND METABOLISM

### Parent toxicity and metabolism

Studies have determined that parabens are weakly estrogenic and can cause other adverse effects (Kasprzyk‐Hordern et al., 2008; Oishi, 2001). The toxicity of these compounds has been shown to increase as lipophilicity increases (Bledzka et al., 2014). Paraben lipophilicity increases with increasing chain length. Evaluated adverse effects include, endocrine disruption, adverse reproductive effects and mitochondrial dysregulation leading to loss of energy and cell death (Tavares et al., 2009). A study by Watanabe et al. (2013) compared paraben agonistic activities with human estrogen receptor α (hERα), human estrogen receptor β (hERβ) and both agonistic and antagonistic activities with the androgen receptor (hAR). The results showed that all parabens were more active with hERβ, and longer chained parabens generally show more activity. No parabens had either agonistic or antagonistic activity towards AR. Some studies have also linked paraben exposure to breast cancer, with more recent studies focusing on breast cancer due to paraben use in underarm personal care products (Darbre & Harvey, 2014). However, research has not yet determined if there is any correlation between paraben use and breast cancer. When evaluating decrease in reproduction rates as a result of parent paraben exposure, benzyl paraben has the highest hazard quotient at 0.25 using a NOEC determined using *Daphnia magna* and a hazard quotient 2.3*10^−4^ determined from a study involving fathead minnow (Yamamoto et al., 2011). This assessment shows that parent paraben species are not likely to have ecological impacts in normal circumstances. However, some areas with high releases or lack of treatment facilities may see concentrations high enough to be significant. Parabens may also induce cell death through ATP depletion. A study by Nakagawa and Moore (1999) found that smaller chained parabens, such as methyl and ethyl parabens exhibited low toxicities but still had decreases in ATP and a 9% increase in cell death when compared with the control. However, toxicity increased with chain length, propyl paraben resulted in a 50% decrease in cell viability and a large decrease in ATP and butyl paraben caused 88% to 98% cell death and a nearly total loss of ATP. This chain length of the compound has a significant impact on mitochondrial toxicity. However, parabens were added to the mitochondrial suspension containing hepatocytes at concentrations ranging from 0.1 to 0.5 mM which is much higher than the concentrations that will be seen in the environment.

In humans, parabens are not expected to accumulate in the body and are mostly excreted in urine. During biotransformation parabens are transformed into sulfate conjugates using sulfotransferases during sulfation and into glucuronide conjugates using UDP glucuronosyltransferases during glucuronidation (Boberg et al., 2010). In humans, paraben metabolism occurs quickly. A pharmacokinetic study of orally administered propyl paraben found that all of the administered dose had been excreted within 72 h (Shin et al., 2019). The majority of the compound has been excreted in urine as a PHBA or para‐hydroxyhippuric acid (PHHA) which is formed when PHBA interacts with glycine. Higher concentrations of conjugated propyl paraben were found in urine when compared with concentrations of free propyl paraben. PHBA and PHHA made up 78% of the total propyl paraben metabolite quantified in urine, conjugates propyl paraben made up 22% and free propyl paraben made up less than 1%. PHBA and PHHA were also excreted faster with all PHBA and PHHA being excreted within 24 h (Shin et al., 2019). Because PHBA and PHHA were present in the highest concentration, it is likely that majority of the administered propyl paraben had been hydrolyzed during biotransformation instead of forming conjugates via sulfonation or glucuronidation. This means propyl paraben used in food will be more likely to be biotransformed to PHBA and PHHA, allowing faster excretion. Similar parabens, such as methyl paraben will likely have similar metabolite fraction when to propyl paraben, biotransformation and excretion of transformation products will vary. Although majority of parent parabens are likely to be excreted quickly as PHBA or PHHA, when ingested, parabens are used more often and at greater concentrations in PCPs than in foods. Therefore, dermal adsorption will be the more common route of paraben exposure. One study found that the majority of dermally administered butyl paraben is excreted within 12 h. However, the amount of butyl parabens recovered in urine was much lower than the amount that had been dermally applied (Janjua et al., 2007). This means that the butyl paraben would have been biotransformed within 12 h. The study did not evaluate conjugated forms of butyl paraben and it is unknown how long the conjugated butyl parabens would remain in the body or how much butyl paraben had been metabolized to PHBA and PHHA. Research into metabolite concentrations after dermal absorption would be useful in determining how much of the compound was adsorbed and biotransformed. One study that evaluated paraben concentrations in urine, serum, and seminal plasma, found that concentrations of methyl, ethyl, propyl, and butyl paraben were much higher in urine than in serum (Frederiksen et al., 2011). This means that a large amount of parent paraben is transformed, excreted, or deposited into tissues rather than remaining in circulation as the parent compound. Although this is true for humans, parabens may not be biotransformation as efficiently in other species. A study that focused on paraben adsorption and excretion in rats found that about 50% of the dermally administered dose was not absorbed and was collected on the carcass after death (Aubert et al., 2012). Urine had the second highest concentration of parabens after dermal exposure and the highest concentration after oral and subcutaneous exposure. Overall, male rats had higher concentrations in urine than female rats except after dermal absorption whereas female rats had higher concentrations in tissues than males. Female rats had overall higher dermal absorption rates, this trend can be seen in humans as well. Studies evaluating urinary concentrations have quantified higher paraben concentrations in females than in males (Smith et al., 2012). This is likely due to dermal adsorption of parabens after use of cosmetics and other PCPs. However, cosmetic use is also a factor to consider when evaluating urinary and tissue concentrations. The increase in paraben concentrations may be due more to cosmetic use rather than faster absorption rates. More research needs to be done to determine dermal adsorption rates in humans. The paper by Bolujoko et al. (2021), provides an in‐depth review on the toxicity of parent paraben compounds.

### Transformation product toxicity

Not much is currently known about transformation product toxicity. Of all the potential paraben disinfection products formed during wastewater treatment, chlorinated parabens have been the most studied. A study by Terasaki, Yasuda, and Shimoi (2015) found that chlorination of parabens reduces aquatic toxicity by evaluating the lowest‐observed‐effect concentration (LOEC) of parent parabens and chlorinated products on offspring production. Methyl paraben, benzyl paraben and dichlorinated benzyl paraben reduced number of offspring significantly. However, chlorinated products had a reduced impact on offspring reduction when compared with their respective parent compounds. This study suggests that chlorination of parabens would reduce paraben toxicity, meaning chlorination would be a potential water treatment method for reducing environmental impacts of parabens and similar compounds. However, a study by Terasaki, Abe, et al. (2015) evaluated chlorinated paraben toxicity in the environment, specifically evaluating their interaction with the aryl hydrocarbon receptor. The results of the study found that longer chained parabens had some activity and all chlorinated parabens, with the exception of chlorinated forms of PHBA and isopropyl paraben had shown activity. Monochlorinated species were potent at lower concentration than their respective dechlorinated forms. This is an unexpected result, in most cases, dichlorinated forms would be expected to have greater activity. However, dichlorinated products will be present in greater concentration in wastewater effluent than the monochlorinated forms due to the speed of the reaction from mono to dichlorinated and the persistence of the dichlorinated form. Therefore, further research should be done to determine if either mono or dichlorinated products would be present in relevant concentrations. Because both mono and dichlorinated PHBA as well PHBA did not have the same agonist activity as the parent compounds, there is a high probability that the hydrolysis of the ester group eliminates the AhR potency or at least greatly reduces the potency (Terasaki, Abe, et al., 2015).

There are not currently any studies that evaluate chlorinated paraben metabolism, it is not known whether the chlorinated products will follow a pattern seen in parent paraben metabolism. Often chlorinated compounds are more difficult to metabolize. Although chlorinated products would be expected to have increased agonistic activity, studies have found that chlorinated parabens reduce or even mask the agonistic activity that has been identified with the parents. However, in a study done by Sasaki and Terasaki (2018), agonist and antagonistic activity of different brominated parabens were evaluated. The results of the study showed that the brominated parabens did not have the same agonist activity as the parents and had even more of a masking effect than shown in chlorinated parabens. Only one of the brominated paraben species, dibrominated benzyl parabens had been determined to be an estrogen agonist However, all monobromated and dibrominated paraben species had an antagonistic effect, where only two non‐brominated species, butyl and benzyl paraben showed antagonistic effects. Increasing chain length and number of bromines both caused an increase in antagonistic activity. This shows that as previously determined, the halogenation of parabens does reduce the agonistic effect, but there is an increasing antagonistic effect.

Some studies have included toxicity of paraben degradation products. A study by Soler de la Vega et al. (2019) found that adding TiO_2_ nanoparticles to mixtures of parabens and UV filters increased toxicity. However, the study did not verify if the increase in toxicity is due to aggregation of TiO_2_ nanoparticles, or to ROS production. However, the authors do note that transformation to more harmful products by ROS may be a factor in increasing toxicity. A study by Gao et al. (2020) also found that the photodegradation of ethyl paraben resulted in increased toxicity in a yeast two‐hybrid reporter assay with photodegradation products of ethyl paraben having higher EC values than that of the ethyl paraben itself. However, the study noted that the increased toxicity was due to formation of oligomers whereas the addition of the OH itself resulted in decreased toxicity. In common treatments, paraben reaction with OH radicals most often result in the addition of OH on the ring, therefore a decrease in toxicity will occur. A study by Ding et al. (2017) found that hydrolysis of parabens using rat liver enzymes resulted in a large decrease in antiandrogenic activities. This means that paraben hydrolysis into PHBA or further degradation into phenols and hydroquinone will result in significantly decreased antiandrogenic activity.

## CONCLUSIONS AND FUTURE PERSPECTIVES

Parabens have been detected in surface water due to their widespread use as preservatives. However, paraben transformation products are detected in greater concentrations than parent parabens. Therefore, research focusing on paraben transformation in water treatments and monitoring of their spread into surface water is necessary to understand the impact parabens have on the environment. Recent scientific findings indicate that transformation of parabens can result in a decrease in toxicity, primarily if transformed to PHBA or hydroxylated products. However, more research would need to be done to verify this and evaluate other toxicological endpoints. Future research directions should focus on identifying treatment products formed in‐situ at water resource recovery facilities and determining the environmental impacts these compounds may have on the environment. Implementation of improved water processes could reduce formation of harmful transformation products. Research focusing on the use of catalysts proves to be a promising method for increasing paraben transformation to potentially less harmful degradation products.

### AUTHOR CONTRIBUTIONS

Michael Penrose reviewed literature, created figures and tables, and wrote the manuscript with input from George Cobb. George Cobb reviewed and revised the manuscript. All authors read and approved the final manuscript.

## Data Availability

All data used in this article were based off results obtained from previous studies, and no new data were created or analyzed in this review. The authors who created the data are cited where the data are included.
